# What is behind partial repetition costs? Event-files do not fully occupy bound feature codes

**DOI:** 10.3758/s13423-023-02253-x

**Published:** 2023-03-03

**Authors:** Viola Mocke, Elena Benini, Juhi Parmar, Moritz Schiltenwolf, Wilfried Kunde

**Affiliations:** 1 Department of Psychology, Julius-Maximilians-Universität, Würzburg, Germany; 2 Institute of Psychology, RWTH Aachen University, Aachen, Germany; 3 Department of Psychology, Friedrich-Schiller-Universität Jena, Jena, Germany; 4 Department of Psychology, Eberhard Karls Universität, Tübingen, Germany

**Keywords:** Event-file, Binding, Code occupation, Partial repetition costs

## Abstract

Feature binding accounts state that features of perceived and produced events are bound into event-files. Performance while responding to an event is impaired when some, as opposed to all or none, of this event's features already belong to a previous event-file. While these partial repetition costs are generally considered to be indicators of feature binding, their cause is still unclear. Possibly, features are fully occupied when bound in an event-file and must be unbound in a time-consuming process before they can enter a novel event-file. In this study, we tested this code occupation account. Participants responded to the font color (target) of a word (distractor) by pressing one of three keys (response) while ignoring the word meaning. We measured partial repetition costs from prime to probe while introducing an intermediate trial. We compared sequences in which this intermediate trial did not repeat any prime features and sequences in which it repeated either the prime response or distractor. Partial repetition costs occurred in the probe, even when one (vs. none) of the prime features repeated in the intermediate trial, although significantly reduced. Thus, single bindings do not fully occupy feature codes. By ruling out a possible mechanism behind partial repetition costs, the present study contributes to the further specification of feature binding accounts.

**Supplementary Information:**

The online version contains supplementary material available at 10.3758/s13423-023-02253-x.

The theory of event coding (TEC; Hommel et al., 2001) states that perceived and produced events are represented by abstract feature codes that can describe both perception and action. For example, seeing a traffic light turning red might activate the feature code “stop,” which is not only the meaning of what you see but also describes your reaction to this percept. According to TEC, such feature codes should not only be activated but also bound to other feature codes describing the same event (e.g., “red”) to minimize confusion with other events. The resulting structure, an “event-file” (Hommel, 2004), can be conceived as a compound of bound feature codes, each describing an attribute of stimuli involved in the event, responses made to these stimuli, or effects these responses produce (Frings et al., 2020; Henson et al., 2014).

A large part of the research on binding processes investigates feature codes relating to responses (R, here: stop) or the (often equivalent) targets prompting these responses (here: red) and feature codes describing irrelevant distractor stimuli (D, for example, the shape of the stop symbol on the traffic light). In one example of such D-R-binding paradigms, a colour-categorization task (Rothermund et al., 2005; see also Frings et al., 2007; Giesen & Rothermund, 2014), participants are asked to recognize the colour of a word while ignoring the word meaning (D) and press a key (R) that has been assigned to this colour.

## Partial repetition costs

Event-file accounts propose that if, for example, in a prime trial, the word *QUIET* appears in *green*, the D-feature *QUIET* should be bound to the R-feature *green*. Typically, responses in subsequent trials (i.e., “probe” trials) are slower and more error prone when one feature repeats from prime to probe trial, but the other feature changes (i.e., partial repetition; e.g. the D-feature changes on R-repetitions or the R-feature changes on D-repetitions) compared with when neither (i.e., no repetition) or both (i.e., full repetition) features repeat (Frings et al., 2007; Hommel, 1998; Rothermund et al., 2005). For example, pressing the *red* key as a response to *QUIET* in the probe trial is more difficult after having responded with the *green* key to *QUIET* in the prime trial than to a different D, such as *SMALL*. This impairment when encountering an event that shares some (as opposed to all or none) of its features with a previous event-file, is usually referred to as partial repetition costs.

## Code occupation

While partial repetition costs are widely used as indicators of feature binding during the prime episode, it remains unclear why they occur (see, however, Weissman et al., 2022, for recent evidence that not only binding but also a signaling mechanism can contribute to these costs). One possibility of how binding produces partial repetition costs is feature code occupation (e.g., Stoet & Hommel, 1999). This idea derives from the proposed reason for why binding is necessary in the first place—namely, to determine which of a limited set of features belong together to make up a current event (e.g., how we know we see a blue shirt and gray pants and not the reverse; Treisman, 1996; Treisman & Schmidt, 1982). One may think of features as brick stones that make up a building. Using a brick in one building makes that brick inaccessible for another building. Consequently, Stoet and Hommel (1999) suggest that when forming an action plan, which they consider to be such an event-file, “integration means occupation, and hence, integrated codes are committed to, or associated with, a particular structure” (p. 1628). They thus proposed that feature codes which are already part of a not-yet-executed action plan are “occupied” until that action plan has been executed. This idea is also in line with the finding that a to-be-memorized feature is less accessible for planning an action which precedes the feature’s recall (Stoet & Hommel, 2002). Similarly, detecting a left or right arrow is impaired when having planned an action requiring the same directional feature (Wühr & Müsseler, 2001). Notably, in all these experiments, the event-files for which binding was tested (i.e., those formed first) were all still required for a later action at the point in time when they partially overlapped with a novel event-file (i.e., the one impaired by partial repetition). Possibly, features are only occupied by an event-file that is still needed in the future. However, a multitude of studies has found partial repetition costs employing sequential designs in which prime event-files did not have to be maintained until probe responses were made (e.g., Frings et al., 2007; Rothermund et al., 2005). Conceivably, bound feature codes remain occupied for a certain amount of time even after the corresponding response has been given. Therefore, bound features might *“no longer be available for representing other events”* (Hommel et al., 2001, p. 836), also during this postaction stage.

Taken together, the code occupation account states that a feature bound in an event-file becomes unavailable for other event-files, meaning that one feature can only be in one event-file at a time. If that were the case, partial repetition costs would probably occur because of an additional processing step necessary in partial but not in full and no repetition trials. This process would be the feature “extraction” (Kikumoto & Mayr, 2020) or the complete “unbinding” (e.g., Brosowsky & Crump, 2018; Koch et al., 2018; Schmidt et al., 2016; Schuch & Koch, 2004; Stoet & Hommel, 1999) of the prime event-file to make the required, repeating feature available for the probe event-file. From a neurophysiological point of view, this could, for instance, mean that cells coding a certain prime feature would change their discharging frequency to synchronize with a new cell assembly coding the probe event (see Engel & Singer, 2001).

In order to bind, for example, the D-feature *QUIET* to the R-feature *red* in the probe, it must first be unbound from the R-feature *green*, to which it might still be bound shortly after the prime response. This unbinding would destroy the prime event-file, so that in all trials following the probe, no partial repetition costs with regards to this prime event-file could occur.

## Code confusion

There are, however, other explanations for partial repetition costs which do not need to rely on code occupation and unbinding. Many have discussed the possibility that partial feature repetition between two events simply leads to code confusion (e.g., Fournier et al., 2014; Fournier et al., 2015; Fournier et al., 2022; Mattson et al., 2012; Mattson & Fournier, 2008). Code confusion may in turn result from the retrieval of a previous event or at least parts of it. As Frings et al. (2020) specified in a recent framework, bound features might be so strongly integrated that encountering one of them again can retrieve the other features bound to it. In other words, encountering an already bound feature “causes the automatic retrieval of a larger part of, or even the whole file, a kind of pattern-completion process that might hamper the creation of new event-files for feature overlapping but non-identical events” (Hommel, 2004, p. 494). To illustrate, during the probe, the activation of the D-feature (*QUIET*), which repeats from the prime, retrieves the bound R-feature from the prime event-file (*green*), which is not required for the probe R and conflicts with the required R *red*. Overcoming such a conflict which occurs in partial but not in full or no repetition trials might then show in partial repetition costs. How exactly such an involuntary retrieval is overcome is not perfectly clear. Unbinding the repeating feature from the previous event-file might thus even in this model be an appropriate means to do so. Other possible mechanisms like suppression or inhibition of the conflicting features however do not imply the deconstruction of partially repeated event-files at all.

## The present experiment

To summarize, the code occupation hypothesis proposes that a feature cannot be part of two events at the same time, thus requiring unbinding in case of partial feature overlap between memorized or otherwise active events and current events. Contrarily, code confusion, or more specifically, feature retrieval accounts, usually state that features of previous events remain bound, whereby through these remaining bindings involuntary retrieval of currently unneeded features can occur.

Deciding between these alternatives is difficult as they offer different explanations for the very same data pattern—that is, partial repetition costs. They differ though, regarding their predictions about whether there should be partial repetition costs with an additional event between prime and probe that contains prime features. The code occupation hypothesis predicts that using such a prime feature destroys the prime event-file: Using a “brick” from a previous event-file dismantles that “building.” Consequently, if code occupation and subsequent unbinding produced partial repetition costs, probe performance should not be affected by the partial repetition of a prime event-file whose features had been intermediately repeated (see Figs. 2 and 3, top left panels). By contrast, the feature retrieval hypothesis allows for intermediate rebinding of prime features while keeping the prime event-file intact thus allowing for prime influences during the probe (i.e., partial repetition costs, see Figs. 2 and 3, bottom left panels).

We investigated whether a feature can only be part of one event-file at any given point in time as assumed in the code occupation hypothesis. In other words, we tested whether, due to unbinding, an event-file is destroyed after one of its features is bound into another event-file.

## Method

We conducted an online sequential priming experiment with an adaptation of a colour categorization task (e.g., Giesen & Rothermund, 2014; Rothermund et al., 2005), which allowed us to assess partial repetition costs between prime and probe trials. As in classic D-R binding paradigms, D-relation and R-relation were varied orthogonally. We modified the original paradigm by introducing an intermediate trial (n−1) which occurred between the prime (n−2) and the probe (n) to test whether a feature can exist in more than one event-file. The n−2➔n−1 D-relation and R-relation as well as the n−1➔n D-relation and R-relation were also orthogonally varied. We employed a paradigm with three R alternatives and a high number of D words to enable conditions in which across three consecutive trials neither R nor D repeat.

We tested for partial repetition costs by comparing trials in which n−2➔n partial feature repetitions did versus did not occur. To foreshadow, we only included sequences with n−1➔n R and D changes (i.e., n−1➔n full alternations) in the analyses. This way, trial n could only repeat features from trial n−2 (or earlier trials), but not from trial n−1, to derive a pure measure of n−2➔n partial repetition costs, which are unaffected by any possible n−1➔n feature repetition.

Previous work has already inserted to-be-ignored stimuli, or trials with a different task between prime and probe (Hommel & Frings, 2020). To our knowledge, this is the first experiment to directly test the influence of an intermediate event-file (n−1) that partially repeats a prime event-file (n−2) on the n−2➔n partial repetition costs using one task.

**Fig. 1:**
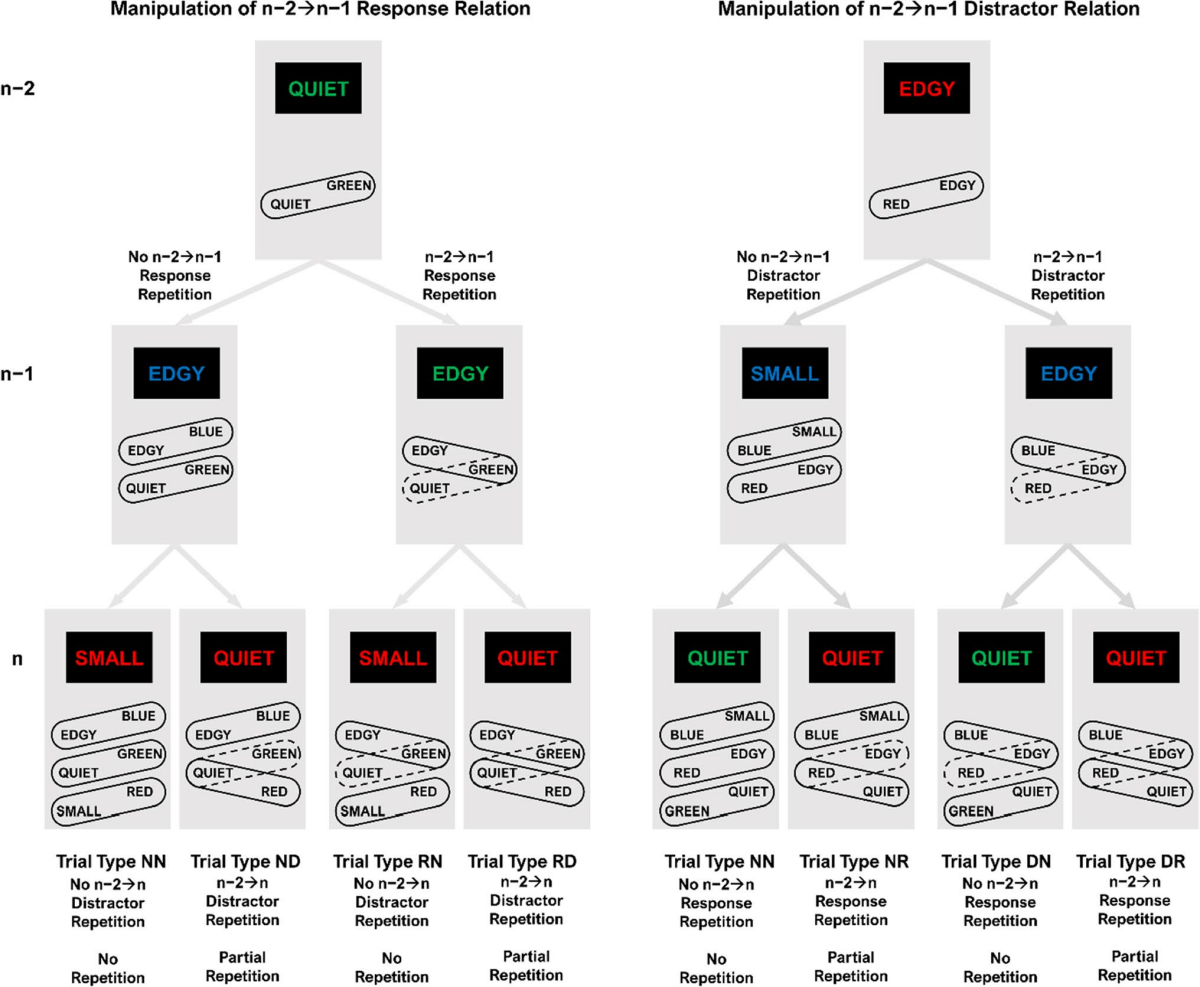
Basic experimental logic for manipulation of n−2➔n−1 response relation and distractor relation. *Note*. The present experiment tested whether the bindings illustrated by dashed lines still exist or whether the respective event-files were destroyed by partial repetition. In any case, there should be partial repetition costs when comparing the two n trials without n−2➔n−1 response repetition (i.e., NN and ND in the left panel) or without n−2➔n−1 distractor repetition (i.e., NN and NR in the right panel). If the dashed bindings persisted, there should also be partial repetition costs in n comparing the two conditions with n−2➔n−1 response repetition (i.e., RN and RD in the left panel) or n−2➔n−1 distractor repetition (i.e., DN and DR in the right panel). Words were presented in German. The first letter of trial type abbreviations reflects the feature repeating from n−2 to n−1 and the second letter the feature repeating from n−2 to n (N: None, D: Distractor, R: Response)

The experimental logic is illustrated in Fig. 1. The first letter of trial type abbreviations reflects the feature repeating from n−2 to n−1 and the second the feature repeating from n−2 to n (N: None, D: Distractor, R: Response). In trial n−2, an event-file consisting of a R- and a D-feature should form. Manipulating n−2➔n−1 feature-relations provides us with the necessary conditions to test the code occupation account. If the code occupation account was correct, there would be unbinding of the n−2 event-file in trial sequences with partial n−2➔n−1 feature repetitions (i.e., trial types RN, RD, DN and DR in Fig. 1). Thus, the n−2 event-file should no longer exist. If the code occupation account was not correct, there would be no unbinding. Thus, the n−2 event-file should still be detectable after a partial n−2➔n−1 feature repetition. Performance in the following trial n should reveal whether the n−2 event-file is still intact. Specifically, n−2➔n partial repetition costs (i.e., RD–RN and DR–DN) should occur if the n−2 event-file was intact, but not if the n−2 event-file was destroyed. Without any n−2➔n−1 feature repetitions (i.e., NN, ND and NR), both code occupation and retrieval accounts predict n−2➔n partial repetition costs (i.e., ND–NN and NR–NN). Therefore, we set out to test the code occupation account by comparing n−2➔n partial repetition costs in trial sequences with partial versus no n−2➔n−1 feature repetitions.

### Participants

A total of *N* = 108 participants were recruited via Prolific from a fluent German population (49 male, 56 female, three diverse, *Mdn*_Age_ = 26, range: 18–61). Informed consent was given by participants before starting the experiment, which was completed on their own desktop devices (85 Win, 22 MacIntel, 1 Linux). The experiment took a median of 24 minutes, for which participants were paid £3.75 according to the payment guidelines by Prolific.

With the same paradigm, Giesen and Rothermund (2014) found a D repetition effect in target and R change trials in reaction times (RTs) sized *d*_*z*_ = 0.39. To replicate this effect with a two-tailed paired-samples *t*-test in the n−2➔n R change condition (i.e., NN vs. ND), with an alpha level of 𝜶 = .05 and a power of 𝜷 = .80, at least 53 participants were required (calculated with G*Power; Faul et al., 2007). However, the main effect of interest in the current study is the 2 × 2 interaction showing that this effect exists without but vanishes in trials with n−2➔n−1 feature repetition (i.e., RN vs. RD). Thus, we estimated that at least twice as many participants would be required (Brysbaert, 2019). For counterbalancing purposes, we collected 108 data sets. This sampling strategy, all hypotheses, as well as experimental design and analysis plans were preregistered (https://osf.io/v3b5j). Preregistrations, raw data, analyses and appendices are available on the Open Science Framework (https://osf.io/n3u2m/).

### Materials

Participants positioned the index, middle, and ring fingers of their right hand on the keys J, K, and L of a QWERTZ keyboard. Each key was assigned one of the colours red, green, or blue and the colour-key mapping was counterbalanced across participants. In each trial, a coloured word was presented, and participants were asked to respond to the font colour of the word (R). D words came from a self-generated pool of 750 two- or three-syllabled German adjectives (see Appendix 1).

### Procedure

Written instructions were followed by a practice block and 15 experimental blocks à 77 trials. Each trial started with the presentation of the D word in white for a random duration of 150, 200 or 250 ms. Then, the word changed into one of the target colours, which stayed on screen for a maximum of 1,500 ms or until a response was given. In case of an incorrect or no response, a grey error message appeared for 500 ms. After this message or after a correct response, an intertrial interval (ITI) of 100 ms followed.

Each of the 75 analyzable trials per block (since the first two trials did not have the necessary preceding n−2 and n−1 trials) served as trial n−2, trial n−1 and trial n. There were 25 possible trial sequences when looking at three trials in a row (i.e., transition combinations from n−2 to n−1 to n; see Appendix 1 for details). The order of trials was determined pseudorandomly by an algorithm, which ensured that all 25 trial sequences appeared exactly 3 times per block. This way, the total experiment with 15 blocks yielded 45 trials per trial sequence.

### Design

The experiment is based on a 2 (n−2➔n−1 feature relation: repetition vs. change) × 2 (n−2➔n feature relation: repetition vs. change) within-subjects design. Dependent measures were RTs and error rates (ERs).

Importantly, we ran two separate analyses to distinguish whether (i) n−2➔n−1 feature relation meant that the R repeated versus changed from n−2 to n−1, and n−2➔n feature relation meant that the D repeated versus changed from n−2 to n (Fig. 1, left panel), or (ii) vice versa (Fig. 1, right panel). In the first case, when we analyzed n−2➔n−1 R relation (with the R-feature potentially destroying event-file n−2), we only included trials with n−2➔n−1 D changes and n−2➔n R changes. In the second case, when we analyzed n−2➔n−1 D relation (with the D-feature potentially destroying event-file n−2), we only included trials with n−2➔n−1 R changes and n−2➔n D changes.

Our first independent variable n−2➔n−1 feature relation describes whether the n−2 event-file could possibly be destroyed by a partial feature repetition in n−1 or not. The second independent variable, n−2➔n feature relation, allowed us to estimate the size of partial repetition costs. Partial repetition costs are often operationalized as the two-way interaction between D-relation and R-relation from prime to probe (e.g., Frings & Moeller, 2012). However, in the present design, if we wanted to include n−2➔n D (R) repetitions in our analysis of the effect of n−2➔n−1 D (R) relation, we would necessarily have to include trials with n−1➔n D (R) repetition. To avoid such n−1➔n feature repetitions, we only included sequences in which the feature that repeated or changed from trial n−2 to n−1 was not the same that repeated or changed from trial n−2 to n. This way, trial n could only repeat features from trial n−2 (or earlier trials), but not from trial n−1. Therefore, trial n could only be affected by partial repetition of n−2 but not of n−1, whose sole purpose was to potentially destroy the n−2 event-file (see Table 1). Consequently, we operationalized partial repetition costs as the difference between one partial repetition condition and the no repetition condition. This approach is commonly used in the action planning literature to quantify partial repetition costs (e.g., Fournier et al., 2015; Fournier & Richardson, 2021; Richardson et al., 2020; Stoet & Hommel, 1999).

**Table 1:** Trial types of interest for both analyses with relations between trials n−2, n−1 and n

Trial Type	n−2➔n−1 Repetition	n−2➔n Repetition	n−1➔n Repetition
No n−2➔n−1 Feature Repetition			
** NN**	**None**	**None**	**None**
** ND**	**None**	**Distractor**	**None**
** NR**	**None**	**Response**	**None**
NB	None	Both	None
n−2➔n−1 Response Repetition			
** RN**	**Response**	**None**	**None**
** RD**	**Response**	**Distractor**	**None**
RR	Response	Response	Response
RB	Response	Both	Response
n−2➔n−1 Distractor Repetition			
** DN**	**Distractor**	**None**	**None**
DD	Distractor	Distractor	Distractor
** DR**	**Distractor**	**Response**	**None**
DB	Distractor	Both	Distractor

*Note*. The first letter of trial type abbreviations reflects the feature repeating from n−2 to n−1 and the second letter the feature repeating from n−2 to n (N: None, D: Distractor, R: Response, B: Both). Bold rows are included in the preregistered analyses and all rows in the post hoc analyses

### Data analysis

For RT and ER analyses, we only considered the relevant trial types and excluded all trials with responses faster than 150 ms, trials that deviated more than 3 standard deviations from the respective RT cell mean and trials n with errors in n−2 and/or n−1 trials. None of the participants had less than 10 correct trials in any experimental cell or more than 30% errors overall.

Mean RTs were calculated from all correct responses per experimental cell, and ERs as the number of all erroneous responses (too slow or incorrect key) divided by all trials per experimental cell.

**Fig. 2:**
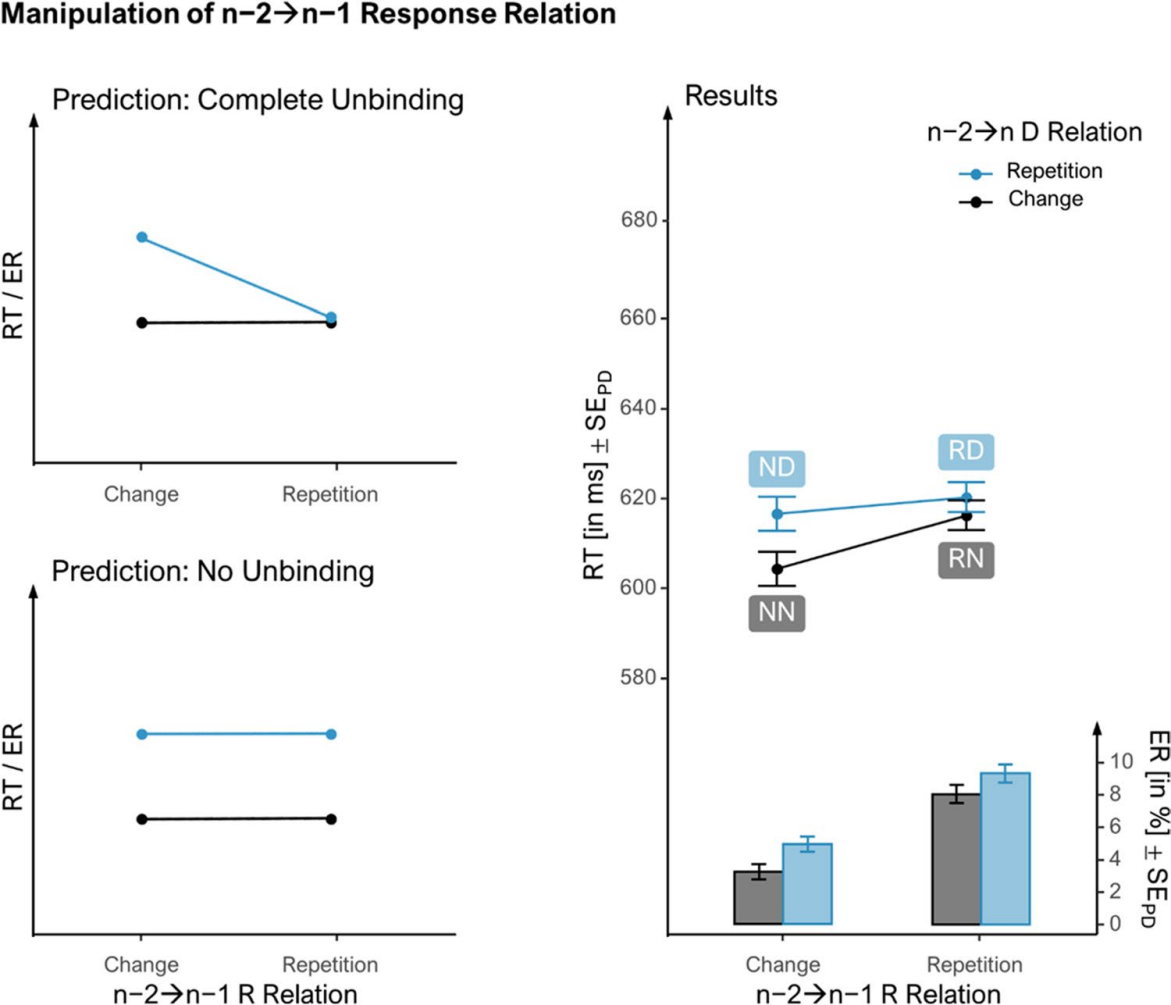
Effect of n−2➔n D-relation by n−2➔n−1 R-relation on reaction times and error rates in *n*. *Note*. The two left panels show possible outcomes for when event-files do (top) or do not (bottom) fully occupy bound feature codes. The right panel shows the effect of manipulating the n−2➔n−1 response (R) relation (while changing the distractor, D) on the effect of the n−2➔n D-relation (while changing the R). Line graphs represent mean reaction times and bar graphs error rates. The first letter of condition labels reflects the feature repeating from n−2 to n−1 and the second letter the feature repeating from n−2 to n (N: None, D: Distractor, R: Response). Error bars represent standard errors of the paired differences (see Pfister & Janczyk, 2013)

We conducted a 2 × 2 repeated-measures ANOVA (rmANOVA) with the within-subjects factors n−2➔n−1 feature relation (repetition vs. change) and n−2➔n feature relation (repetition vs. change) for each feature that could repeat from n−2 to n−1 (D vs. R) and each dependent measure (four rmANOVAs in total). We additionally tested individual partial repetition costs separately for the two levels of n−2➔n−1 feature relation with follow-up two-tailed *t*-tests. The standard errors of the paired differences are depicted as error bars in Fig. 2 (Pfister & Janczyk, 2013). We also decided post hoc to provide Bayes factors for the 2 × 2 interactions and their interpretations (Jeffreys, 1961).

Table 1 (Bold rows) presents the seven trial sequences of interest also depicted in Fig. 1. For both analyses described below, trial type NN (i.e., those sequences without n−2➔n−1 or n−2➔n feature repetition) served as a baseline for the calculation of the partial repetition costs without n−2➔n−1 feature repetition.

#### Manipulation of n−2➔n−1 R-relation

The costs of n−2➔n D-feature repetitions (while the R-feature changed) were computed as the performance (RTs and ERs) difference between n−2➔n D repetitions and changes. We examined whether these costs were moderated by n−2➔n−1 R-relation (repetition vs. change) while the D-feature changed. Labelling the conditions as in Table 1, the interaction in the rmANOVA tests whether the costs of n−2➔n D-feature repetitions without n−2➔n−1 feature repetition (ND-NN) minus the same costs but with n−2➔n−1 R repetition (RD-RN) was significantly different from 0.

#### Manipulation of n−2➔n−1 D-relation

Likewise, costs of n−2➔n R-feature repetitions (while the D-feature changed) were computed as the performance difference between n−2➔n R repetitions and changes. We examined whether these costs were moderated by n−2➔n−1 D-relation while the R-feature changed. The interaction in the rmANOVA tests whether the costs of repeating the R-feature (vs. no feature) from n−2 to n without n−2➔n−1 feature repetition (NR-NN) minus the same costs but with n−2➔n−1 D repetition (DR-DN) is significantly different from 0.

## Results

### Manipulation of n−2➔n−1 R-relation

The right panel of Fig. 2 shows the effect of n−2➔n−1 R-relation on the effect of n−2➔n D-Relation, that is, the costs of repeating the D from n−2 in n. Responses were generally slower with n−2➔n−1 R-repetition than without, *F*(1, 107) = 8.08, *p* = .005 , η_p_^2^ = .07*,* η_G_^2^ < .01. Also, responses were slower with n−2➔n D-repetition than without, *F*(1, 107) = 9.02, *p* = .003 , η_p_^2^ = .08*,* η_G_^2^ < .01, reflecting overall partial repetition costs (*d*_*z*_ = 0.29). While the costs were significant with n-2➔n-1 R-changes, *M* = 12, *t*(107) = 3.24, *p* = .002, *d*_*z*_ = 0.31, they failed to reach significance with n-2➔n-1 R-repetitions, *M* = 4, *t*(107) = 1.22, *p* = .225, *d*_*z*_ = 0.12. Nonetheless, the two-way interaction, that is, the difference between these partial repetition costs failed to reach significance, *F*_(1,107)_ = 3.12, *p* = .080 , η_p_^2^ = .03*,* η_G_^2^ < .01 (anecdotal evidence for null hypothesis, BF_01_ = 2.09).

Responses were less accurate with n−2➔n−1 R-repetition than without, *F*(1, 107) = 91.25, *p* < .001 , η_p_^2^ = .46*,* η_G_^2^ = .14. The ERs also reflected partial repetition costs overall, *F*(1, 107) = 17.23, *p* < .001, η_p_^2^ = .14*,* η_G_^2^ = .02 (*d*_*z*_ = 0.40). Importantly, the interaction was again not significant, *F*(1, 107) = 0.35, *p* = .557 , η_p_^2^ < .01*,* η_G_^2^ < .01 (moderate evidence for null hypothesis, BF_01_ = 7.91). Costs were significant both when the R changed from n−2 to n−1, *M* = 1.7, *t*(107) = 3.63, *p* < .001, *d*_*z*_ = 0.35, and when it repeated, *M* = 1.3, *t*(107) = 2.27, *p* = .026, *d*_*z*_ = 0.22.

### Manipulation of n−2➔n−1 D-relation

**Fig. 3:**
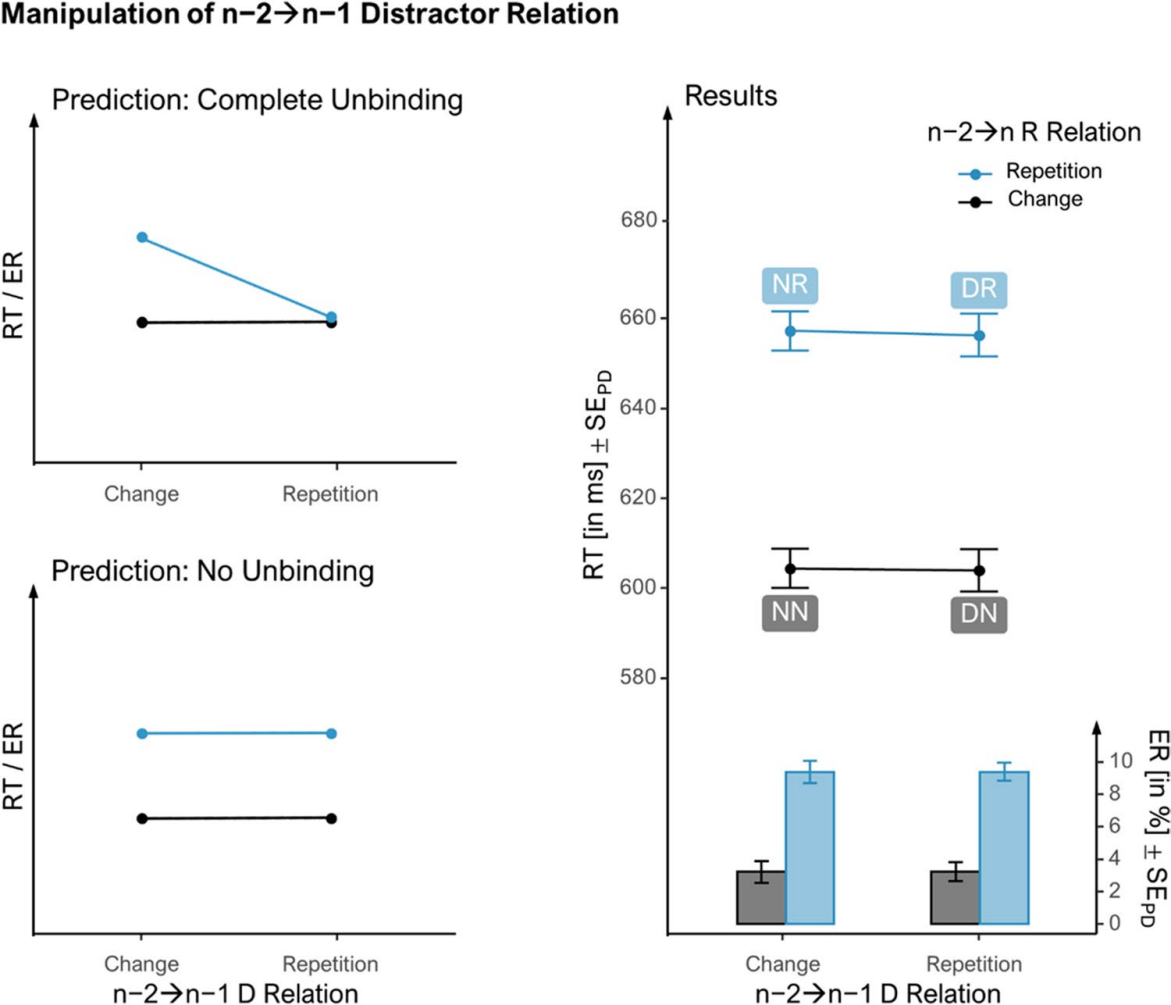
Effect of *n−2➔n* R-relation by *n−2➔n−*1 D-relation on reaction times and error rates in *n*. *Note*. The two left panels show possible outcomes for when event-files do (top) or do not (bottom) fully occupy bound feature codes. The right panel shows the effect of manipulating the n−2➔n−1 distractor (D) relation (while changing the response, R) on the effect of the n−2➔n R-relation (while changing the D). Line graphs represent mean reaction times and bar graphs error rates. The first letter of condition labels reflects the feature repeating from n−2 to n−1 and the second letter the feature repeating from n−2 to n (N: None, D: Distractor, R: Response). Error bars represent standard errors of the paired differences (see Pfister & Janczyk, 2013)

When manipulating the n−2➔n−1 D-relation (Fig. 3, right panel), there was no main effect of n−2➔n−1 D-relation in RTs, *F*(1, 107) = 0.06, *p* = .800 , η_p_^2^ < .01*,* η_G_^2^ < .01. Responses were slower with n−2➔n R-repetition than without, *F*(1, 107) = 212.02, *p* < .001 , η_p_^2^ = .67*,* η_G_^2^ = .08, reflecting overall partial repetition costs (*d*_*z*_ = 1.40). Importantly, these costs were not significantly larger with n−2➔n−1 D-changes, *M* = 52.86, *t*(107) = 12.18, *p* < .001, *d*_*z*_ = 1.17, than with n−2➔n−1 D-repetitions, *M* = 52.32, *t*(107) = 11.02, *p* < .001, *d*_*z*_ = 1.06, as there was no interaction, *F*(1, 107) = 0.01, *p* = .923 , η_p_^2^ < .01*,* η_G_^2^ < .01 (moderate evidence for null hypothesis, BF_01_ = 9.33).

ERs in n did not differ between n−2➔n−1 D-relation conditions, *F*(1, 107) < 0.01, *p* < .964 , η_p_^2^ < .01*,* η_G_^2^ < .01, but they reflected partial repetition costs, *F*(1, 107) = 158.84, *p* < .001, η_p_^2^ = .60*,* η_G_^2^ = .24 (*d*_*z*_ = 1.21). These were however not larger with n−2➔n−1 D-changes, *M* = 6.16, *t*(107) = 9.16, *p* < .001, *d*_*z*_ = 0.88, than with n−2➔n−1 D-repetitions, *M* = 6.16, *t*(107) = 10.82, *p* < .001, *d*_*z*_ = 1.04, as shown by the interaction term, *F*(1, 107) < 0.01, *p* = .995 , η_p_^2^ < .01*,* η_G_^2^ < .01 (moderate evidence for null hypothesis, BF_01_ = 9.37).

## Discussion

### Manipulation of n−2➔n−1 R-Relation

We tested the idea that a feature can only be in one event-file at a time by investigating whether partial repetition costs in a trial n with respect to n−2 would vanish if parts of the n−2 event-file reoccurred in n−1. First, we looked at whether the n−2 event-file was destroyed by a repeating R (but changing D) in the n−1 event-file. Neither in RTs nor ERs did we find a significant modulation of the costs of partially repeating the n−2 D-feature in n by the n−2➔n−1 R-relation. More precisely, the costs of n−2➔n D-repetition were descriptively but not significantly smaller with n−2➔n−1 R-repetition than R-change. This is evidence against the full code occupation hypothesis. Another interesting aspect is that with n−2➔n−1 R-repetition (vs. change), both measures yielded hampered performance in n (where the required R would always change). Possibly, when the two preceding trials required the same R, this R still had a particularly high activation level in trial n, making it more difficult to execute a different R than when the n−2 R had not been repeated in n−1.

### Manipulation of n−2➔n−1 D-relation

Second, we looked at whether the n−2 event-file was destroyed by a repeating D in n−1, while the R changed. Similarly, we did not find a significant modulation of the costs of partially repeating the n−2 R-feature in n by the n−2➔n−1 D-relation. That is, the costs of n−2➔n R-repetition were not smaller with n−2➔n−1 D-repetition than D-change. This, again, does not support the full code occupation hypothesis. In contrast to the R-feature, n−2➔n−1 D-repetition (vs. change), did not result in hampered performance. Arguably, this could be due to the D-feature being task irrelevant.

### Partial repetition costs as difference between partial and no repetition trials

We had operationalized partial repetition costs as the performance difference between partial and no feature repetition conditions. Strikingly, overall costs of repeating only the n−2 R-feature in n versus no feature (*d*_*z*_ = 1.40 for RTs, *d*_*z*_ = 1.21 for ERs), were much larger than the costs of repeating only the D-feature versus no feature (*d*_*z*_ = 0.29 for RTs, *d*_*z*_ = 0.40 for ERs). Regarding the costs of repeating only the R-feature, one might find that the hampered performance in partial repetition trials (from n−2 to n) compared with no repetition trials might not be caused by binding alone, but by a more general bias to not repeat the n−2 R in n. Possibly, the intermediate R or perhaps even only a longer delay between prime and probe might have produced this bias, favouring R change trials (see Druey, 2014; Hübner & Druey, 2006; Pashler & Baylis, 1991).

However, we argue that this account alone cannot explain partial repetitions costs in our data. First, such an account would only explain n−2➔n partial repetition costs of the R (see right panel in Fig. 1), but not of the D, because here the R never repeated from n−2 to n (see left panel in Fig. 1). Second, we used a three-choice task. Thus, any tendency to not repeat a previous R would not come with preparation of a specific alternative response, as there were two of them.

### Post hoc analyses

**Fig. 4:**
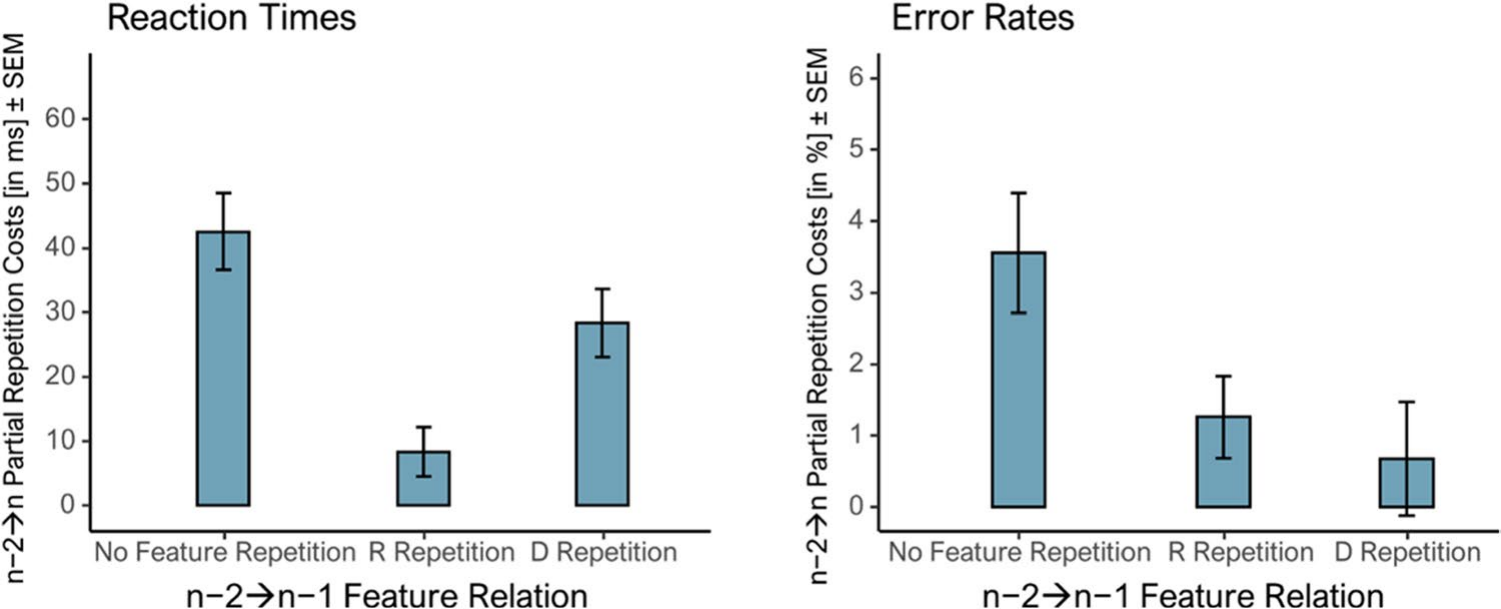
Size of *n*−2➔*n* partial repetition costs by *n*−2➔*n*−1 feature relation. *Note*. Partial repetition costs, measured as the size of the 2 (distractor, D, relation) × 2 (response, R, relation) interaction for reaction times (left panel) and error rates (right panel) between trial n−2 and n. Costs were significant in RTs and ERs when no or the R feature repeated from n−2 to n−1 and for RTs when the D feature repeated from n−2 to n−1. Error bars represent individual standard errors of the means

Still, to exclude the possibility that partial repetition costs when manipulating n−2➔n−1 D-relation were the result of a general response alternation bias, we conducted post hoc analyses (see Appendix 2 and 3), operationalizing partial repetition costs as the two-way interaction between D-relation and R-relation from prime to probe (i.e., using all rows in Table 1; Frings & Moeller, 2012; Frings et al., 2007). Figure 4 shows the n−2➔n partial repetition costs without n−2➔n−1 feature repetition (*M* = 43ms, *M* = 3.6%), when the R repeated (*M* = 8ms, *M* = 1.3%), and when the D repeated (*M* = 28ms, *M* = 0.7%). Crucially, with this different operationalization too, partial repetition costs were significant in at least one measure irrespective of n−2➔n−1 feature repetition, thus leading to the same conclusion as the preregistered analyses without n−1➔n feature repetition.

## General discussion

### Evidence against full code occupation

We had predicted that if a feature can only be in one event-file at a time, we would observe partial repetition costs in trial n with respect to n−2 when no feature repeats from n−2 to n−1, but not when one feature repeats from n−2 to n−1, as this feature repetition should destroy the n−2 event-file. This is not what we observed, as we also found partial repetition costs when either the n−2 R or D repeated in n−1. Therefore, we can refute a strict code occupation account that predicts that one feature can only be in one event-file at a time and that an event-file is destroyed when one of its features is rebound.

This is in line with previous studies that had already provided some indications against code occupation. For instance, code occupation does not predict a difference in the ease of unbinding an action feature from an event-file depending on the feature’s serial position in the action sequence. Still, several studies on action sequences yielded larger partial repetition costs regarding earlier than later features of an action sequence (e.g., Fournier et al., 2014; Mattson et al., 2012; O'Seaghdha & Marin, 2000; Sevald & Dell, 1994). Also, in an action planning paradigm, Mattson and Fournier (2008) found that a distractor can activate an associated response, even when this distractor was already part of a prepared action plan. That is, features bound in not-yet-executed action files are not encapsulated or occupied in a way that they become completely inaccessible. The authors argued that partial repetition costs should therefore arise not at a response activation but at a response selection level, which is more in line with code confusion than occupation.

### The binding problem

While, at first glance, it seems very economical that features can be part of multiple event-files at the same time it brings about theoretical issues. Binding has been proposed to be necessary to determine which features go together at any given point in time (Treisman, 1996; Treisman & Schmidt, 1982). If event-files of previous episodes remain despite rebinding of their features, the very problem to determine which feature belongs to which episode obviously becomes harder. How is it possible to distinguish a present event from past or anticipated future events with the same features? Event-files of present episodes might be qualitatively different from those of memorized or anticipated episodes (e.g., episodic vs. long-term bindings; Colzato et al., 2006). Also, to reduce code confusion, partially overlapping events might be made more distinguishable from each other by weighting those features that are distinct more heavily than shared features (Fournier et al., 2022; see Cox & Criss, 2020, p.37, for related memory models). What we consider also possible, however, is that features of current events are bound more strongly than features from previous episodes. Assuming that binding strength reflects the recency or relevance of an event, it should be possible to flexibly change binding strengths to fit the current goals. Moreover, if a feature is strongly bound to another, partial unbinding (as opposed to full unbinding as studied here) might be necessary to assign even more binding strength to a more relevant link with another feature. In line with this idea of a limited binding capacity, Oberauer (2019) reasoned that working memory capacity might reflect a limit on bindings that can be maintained simultaneously. If so, this would ask for a redescription of the binding problem: The question is not which features are bound with each other to make up a currently needed event-file but rather, which features are bound more strongly with each other than with other possible features.

To conclude, the present study shows that event-files remain integrated even when features of these files enter new files later. Thus, it remains unclear how the cognitive system distinguishes current and previous episodes—a problem that needs to be addressed by future binding accounts.

## Supplementary Information

ESM 1 (PDF 323 kb)

## Data Availability

Preregistrations, raw data, analyses and supplementary material are available on the Open Science Framework (https://osf.io/n3u2m/). The sampling strategy, all hypotheses, as well as experimental design and analysis plans were preregistered (https://osf.io/v3b5j).
