# Supplementary material for: What is behind partial repetition costs? Event-files do not fully occupy bound feature codes
Source: Psychon Bull Rev. 2023 Mar 3;30(4):1463–74. doi: 10.3758/s13423-023-02253-x (PMC10482800; doi:10.3758/s13423-023-02253-x)
Supplement: Supplementary file 1 — (PDF 323 kb) [file 13423_2023_2253_MOESM1_ESM.pdf]

Appendices for

What Is Behind Partial Repetition Costs? Event-files Do Not Fully Occupy Bound Feature Codes

Viola Mocke[1], Elena Benini[2], Juhi Parmar[3], Moritz Schiltenwolf[4], and Wilfried Kunde[1]

[1]Department of Psychology, Julius-Maximilians-University of Würzburg, Germany

[2]Institute of Psychology, RWTH Aachen University, Germany

[3]Department of Psychology, Friedrich Schiller University Jena, Germany

[4]Department of Psychology, Eberhard Karls University of Tübingen, Germany

Appendix A

Table A

Possible Transitions in Sequences of Three Trials in a Row for D and R Features.

Trial Sequence	n−1 to n	n−2 to n	n−2 to n−1
1	Repetition	Repetition	Repetition
2	Repetition	Change	Change
3	Change	Repetition	Change
4	Change	Change	Repetition
5	Change	Change	Change

List of Distractor Words

abnorm, abrupt, abstrus, absurd, achtbar, achtsam, adlig, adrett, affig, affin, aktiv, albern, allein, altklug, amtlich, antik, apart, arglos, armlang, artig, astrein, autark, banal, bange, barfuß, barock, barsch, basisch, bauchig, beblümt, bedeckt, befugt, begabt, behaart, beheizt, beherzt, beige, beißend, bekannt, belebt, belegt, beliebt, benutzt, bepelzt, bequem, bereit, bergig, berstig, berühmt, besorgt, betagt, betont, betrübt, betucht, beugbar, bewährt, bewegt, bewehrt, bewölkt, bewusst, bieder, billig, binär, bissig, bitter, bizarr, blank, blass, bleich, bleiern, blind, blond, blumig, bombig, borstig, boshaft, brach, breiig, breit, brisant, bronzen, brünett, buchen, bucklig, bündig, bullös, chillig, clever, cremig, dänisch, dankbar, debil, defekt, dement, demütig, denkbar, dental, derbe, devot, dezent, dicht, diesig, diffus, direkt, diskret, divers, doppelt, drahtig, drall, dreckig, drehbar, dreist, dröge, drollig, dürftig, düster, dufte, duldsam, dumpf, dunkel, durstig, dusslig, dutzend, eckig, ehrbar, ehrlich, ehrlos, eifrig, eigen, eilig, einfach, einig, einsam, einstig, einzeln, einzig, eisern, eisfrei, eisig, eiskalt, eitel, eklig, elend, empört, endlich, endlos, enorm, entzwei, episch, erblich, erdnah, erneut, ernst, erpicht, essbar, ethisch, exakt, expert, extern, extinkt, extrem, fähig, fällig, fahrend, fahrig, famos, farbig, farblos, fatal, feige, fertig, fettig, fetzig, feudal, feurig, fiebrig, fiktiv, final, findig, finster, flach, fleißig, flink, flippig, flott, flügge, flüssig, fluffig, folgend, folgsam, formal, formell, formlos, forsch, forte, fossil, fragil, frech, fremd, frisch, fromm, frontal, frostig, früher, füllig, gängig, galant, gallig, garstig, gasig, gedeckt, gefasst, geheim, geheist, geistig, geizig, gelaunt, geleckt, gelehrt, gemäß, gemein, genau, geneigt, genial, geraum, gerecht, gering, gerollt, gesamt, gestrig, gesucht, gesund, getreu, getrost, gewandt, gewillt, gewiss, gewohnt, gezielt, giftig, gläsern, glasig, glatt, gleich, global, gnädig, golden, goldig, gotisch, gottlos, grätig, grantig, gratis, grausam, grazil, grell, griffig, grimmig, grotesk, grottig, gültig, günstig, gütig, haarig, hämisch, häufig, hager, haltbar, haltig, happig, harmlos, harsch, hastig, heftig, heikel, heilig, heillos, heiser, heiter, helle, herzlos, hiesig, hitzig, höflich, hölzern, hörbar, holprig, hübsch, hungrig, hurtig, hybrid,

immens, immun, innig, instant, intakt, intern, intim, irdisch, jährig, jetzig, jubelnd, kaputt, kariert, kausal, kehlig, kernlos, keusch, kiefern, kitzlig, klamm, klasse, klebrig, klein, klobig, knackig, knapp, knollig, knorrig, knuffig, kokett, komisch, kommend, kompakt, komplex, konform, konfus, konkav, konkret, konträr, konvers, konvex, korkig, korrekt, kräftig, krumm, künftig, kulant, kundig, kupfern, kurrent, kursiv, labil, lässig, lästig, läufig, langsam, lasch, lasziv, latent, launig, lausig, lauter, lautlos, lauwarm, lebhaft, leblos, lecker, ledern, ledig, legal, leger, leicht, leidsam, leise, lesbar, letal, licht, liniert, listig, livid, locker, löblich, logisch, lokal, loyal, lüstern, lunar, lustig, lustlos, luzid, lyrisch, machbar, madig, mächtig, mäßig, mager, magisch, malad, malisch, manisch, manuell, markant, marmorn, massig, massiv, meliert, mental, messbar, mickrig, milde, mobil, modal, modern, modrig, möglich, mollig, mondän, mondlos, morbid, morgig, morsch, mühsam, mündig, mürbe, müßig, muffig, mukös, mulmig, mundtot, munter, musisch, mutig, mutlos, nackt, nämlich, nasal, nativ, neblig, neidlos, nervig, nervös, neutral, nichtig, nieder, niedrig, nobel, nötig, normal, nutzbar, nutzlos, obskur, obszön, örtlich, östlich, offen, offline, okkult, online, optisch, paarig, packend, pampig, papiern, parat, passend, passiv, patent, patzig, pelzig, perfekt, perplex, pervers, pfiffig, pieksig, pikant, pikiert, platt, pleite, plump, pompös, positiv, postum, potent, präsent, präzis, prall, prekär, prima, primär, privat, probat, profan, profund, prollig, promisk, prompt, proper, prüde, pulvrig, putzig, quälend, quirlig, quitt, radial, räudig, rahmig, randlos, ranzig, rasant, rasch, rasend, rastlos, ratsam, rauchig, recht, reell, reglos, reich, reizbar, reizend, reizlos, restlos, reuig, richtig, riesig, riskant, rissig, robust, römisch, rollig, rosig, rossig, rostig, royal, ruchlos, rührig, ruhig, ruppig, rußig, sacht, sämig, sahnig, salopp, salzig, samten, samtig, sandig, sauber, sauer, schade, schäbig, schal, scharf, scheu, schick, schief, schier, schlaff, schlank, schlau, schmal, schnell, schön, schroff, schwach, schwer, schwül, sediert, seicht, seiden, seidig, selig, selten, seltsam, sicher, simpel, sinnig, sinnlos, sittsam, skurril, solar, solid, sonnig, sonstig, sorglos, sparsam, speckig, spontan, sportiv, spürbar, spurlos, stabil, stämmig, ständig, stark, starr, staubig, steil, steinig, steril, stetig, stickig, still, stoisch, stolz, straff, strikt, stumm, stutzig, subtil, süchtig, südlich, süffig, süßlich, sulzig, super, suspekt, täglich, tätig, taktil, taktlos, tanzbar, tapfer, teilbar, teuer, tönern, tonisch, torlos, total, träge, tragbar, traurig, traut, trendig, triftig, trocken, trotzig, trübe, tüchtig, typisch, üblich, übrig, üppig, ulkig, unbar, undicht, uneins, ungut, unklar, unreif, unschön, unstet, untreu, unwahr, uralt, urban, urbar, valid, vegan, venös, verbal, verpönt, versaut, viral, viril, viskos, vital, völlig, vorig, vornehm, wachsam, wacker, wässrig, wegsam, wehrig, wehrlos, weich, weinig, weise, wenig, wertlos, wichtig, widrig, willig, windig, winzig, wirksam, witzig, wolkig, wortlos, würdig, würflig, würzig, wütend, zaghaft, zedern, zeitig, zeitlos, zeitnah, zickig, zinnern, zivil, zornig, züchtig, zügig, zünftig, zugig, zwirnen, stramm, löslich, lockig, deckend, zynisch, vulgär, luftig, zinslos, spitz, gereizt, zentral, ratlos, zackig, sperrig, maßvoll, dämmrig, dämlich, krank, brutal, robst, sanft, aasig, fidel, flächig, fokal, sakral, freudig, bärtig, behuft, basal, dunstig, schräg, milchig, erdig, erste, zweite, dritte, vierte, fünfte, sechste, siebte, achte, neunte, zehnte, elfte, zwölfte, wertig, kantig, steif, spritig, fesch

Appendix B

Partial Repetition Costs as Two-Way Interaction

Traditionally, partial repetition costs in DRB paradigms are measured as a 2 (D prime-probe relation) x 2 (R prime-probe relation) interaction (Frings & Moeller, 2012; Frings et al., 2007). We decided to conduct post hoc analyses, operationalizing partial repetition costs this way.

Data exclusion criteria were the same as for the previous analyses. Again, none of the participants had less than 10 correct trials in any experimental cell.

Feature Relation between n−2 and n

We aimed to quantify partial repetition costs in trial n with regard to trial n−2. We now also included trial sequences with feature repetitions from n−1 to n. As before, three different types of feature relations between n−2 and n−1 were of interest, that is, no feature repetition, R repetition, and D repetition.

In line with our research question, we tested the moderating effect of feature relation between n−2 and n−1 (No feature repetition vs. R repetition vs. D repetition) on the partial repetition costs in n with respect to n−2. To simplify the analysis, for each of the three conditions, we separately quantified partial repetition costs as the difference between the differences (Frings & Moeller, 2012).

These costs were compared using a one-way rmANOVA with the independent variable feature relation between n−2 and n−1 (No feature repetition vs. R repetition vs. D repetition) and follow-up paired-samples *t*-tests.

Results

Figure B shows the two-way interactions for each of the three conditions. The one-way rmANOVA for partial repetition costs with the independent variable feature relation between n−2 and n−1 (No feature repetition vs. R repetition vs. D repetition) turned out significant for RTs, $F(1.85,198.48) = 10.24$, $p < .001$, $\eta^2_p = .09$, $\eta^2_G = .07$, and ERs, $F(1.76,188.60) = 3.83$, $p = .028$, $\eta^2_p = .04$, $\eta^2_G = .03$ (Greenhouse-Geisser correction for degrees of freedom).

When the R repeated from n−2 to n−1 (M = 8ms, M = 1.3%), costs were smaller than when no feature repeated (M = 43ms, M = 3.6%), for RTs, $t(107) = 4.64$, $p < .001$, $d_z = 0.45$, and ERs, $t(107) = 2.23$, $p = .028$, $d_z = 0.21$. When the D repeated from n−2 to n−1 (M = 28ms, M = 0.7%), they were descriptively, for RTs, $t(107) = 1.66$, $p = .100$, $d_z = 0.16$, and significantly for ERs, $t(107) = 2.25$, $p = .027$, $d_z = 0.22$, smaller than when no feature repeated. When the R repeated from n−2 to n−1, partial repetition costs were significantly smaller than when the D repeated, for RTs, $t(107) = 2.96$, $p = .004$, $d_z = 0.29$, but did not differ for ERs, $t(107) = -0.61$, $p = .545$, $d_z = 0.06$.

Figure B

Effect of D- and R-Relation between n−2 and n by Feature Relation between n−2 and n−1

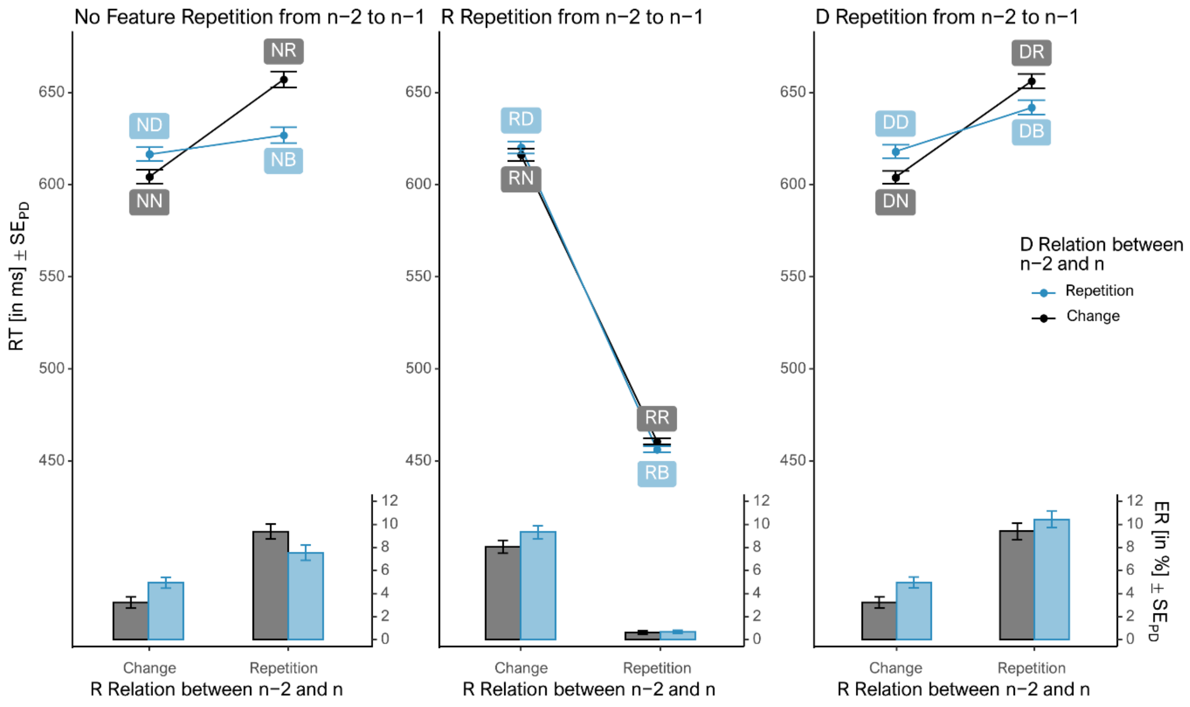

Note. Line graphs represent mean reaction times and bar graphs error rates. The three panels show the effect of response (R) relation and distractor (D) relation between n−2 and n while no feature (left), only the R-feature (middle) or only the D-feature (right) repeated from n−2 to n−1. The first letter of condition labels reflects the feature repeating from n−2 to n−1 and the second letter the feature repeating from n−2 to n (N: None, D: Distractor, R: Response). Half of the conditions of each panel entered the pre-registered analysis shown in Figure 2. Error bars represent standard errors of the paired differences (see Pfister & Janczyk 2013).

Appendix C

Feature Relation between n−1 and n

As a second exploratory analysis, we repeated the approach from Appendix B but for partial repetition costs in trial n with regard to trial n−1 (instead of n−2). Importantly, and differently to previous DRB studies, we examined whether the size of these partial repetition costs depended on whether no feature, the R, or the D repeated from trial n−2 to n−1.

Again, to simplify the analysis, for each of the three conditions, we separately quantified partial repetition costs. These costs were compared using a one-way rmANOVA with the independent variable feature relation between n−2 and n−1 (No feature repetition vs. R repetition vs. D repetition) and follow-up paired-samples *t*-tests. The full 3 x 2 x 2 rmANOVA is presented in the Appendix B.

Results

Figure C shows the two-way interactions for each of the three conditions. The one-way rmANOVA for partial repetition costs with the independent variable feature relation between n−2 and n−1 (No feature repetition vs. R repetition vs. D repetition) turned out significant for RTs, $F_{(1.98,211.95)} = 45.93$, $p < .001$, $\eta^2_p = .30$, $\eta^2_G = .20$, and ERs, $F_{(1.94,207.21)} = 11.79$, $p < .001$, $\eta^2_p = .10$, $\eta^2_G = .06$ (Greenhouse-Geisser correction for degrees of freedom).

When the R repeated from n−2 to n−1 (M = 22ms, M = 1.9%), costs were smaller than when no feature repeated (M = 74ms, M = 6.04%), for RTs, $t(107) = 9.19$, $p < .001$, $d_z = 0.88$, and ERs, $t(107) = 4.44$, $p < .001$, $d_z = 0.43$. When the D repeated from n−2 to n−1 (M = 68ms, M = 4.4%), partial repetition costs were not smaller than when no feature repeated, for RTs, $t(107) = 1.04$, $p = .303$, $d_z = 0.10$, but they were for ERs, $t(107) = 2.02$, $p = .046$, $d_z = 0.19$. When the R repeated from n−2 to n−1, partial repetition costs were significantly smaller than when the D repeated, for RTs, $t(107) = 7.70$, $p < .001$, $d_z = 0.74$, and ERs, $t(107) = 3.04$, $p = .003$, $d_z = 0.29$.

Figure C

Effect of D- and R-Relation between n−1 and n by Feature Relation between n−2 and n−1

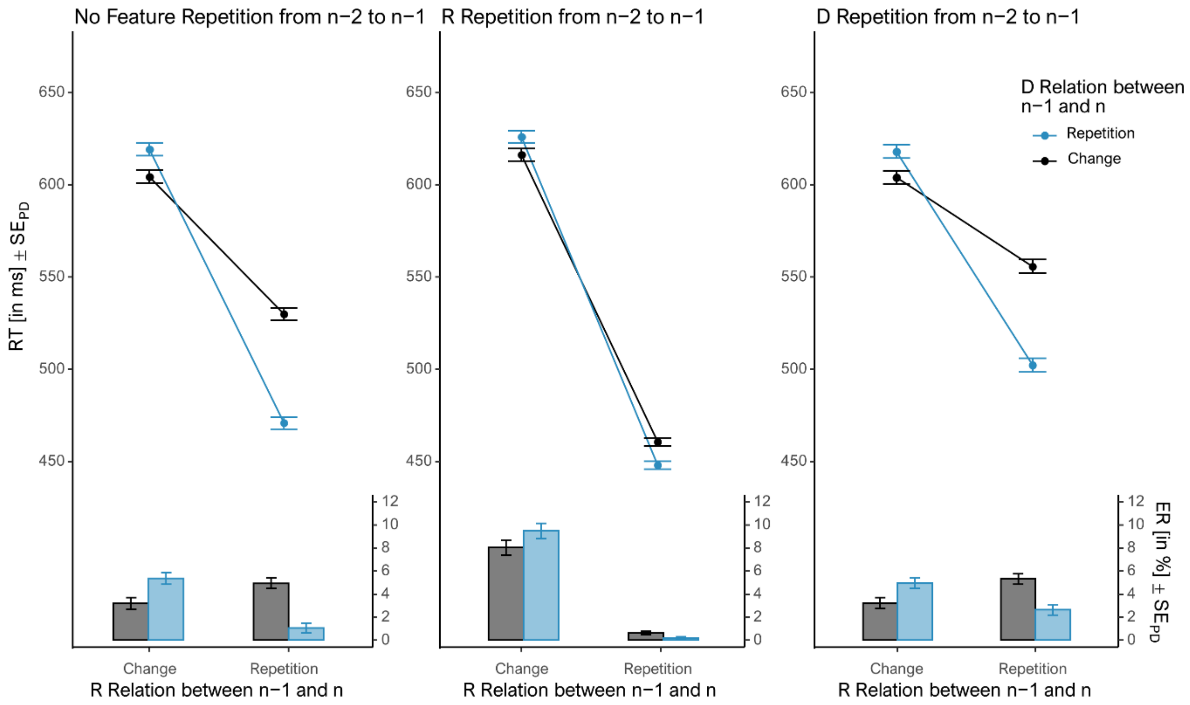

Note. Line graphs represent mean reaction times and bar graphs error rates. The three panels show the effect of response (R) relation and distractor (D) relation between n−1 and n while no feature (left), only the R-feature (middle) or only the D-feature (right) repeated from n−2 to n−1. Error bars represent standard errors of the paired differences (see Pfister & Janczyk 2013).
